# Fish as Reservoirs and Vectors of *Vibrio cholerae*

**DOI:** 10.1371/journal.pone.0008607

**Published:** 2010-01-06

**Authors:** Yigal Senderovich, Ido Izhaki, Malka Halpern

**Affiliations:** 1 Department of Evolutionary and Environmental Biology, Faculty of Science and Science Education, University of Haifa, Mount Carmel, Haifa, Israel; 2 Department of Biology Education, Faculty of Science and Science Education, University of Haifa, Oranim, Tivon, Israel; Columbia University, United States of America

## Abstract

*Vibrio cholerae*, the etiologic agent of cholera, is autochthonous to various aquatic environments, but despite intensive efforts its ecology remains an enigma. Recently, it was suggested that copepods and chironomids, both considered as natural reservoirs of *V. cholerae*, are dispersed by migratory waterbirds, thus possibly distributing the bacteria between water bodies within and between continents. Although fish have been implicated in the scientific literature with cholera cases, as far as we know, no study actually surveyed the presence of the bacteria in the fish. Here we show for the first time that fish of various species and habitats contain *V. cholerae* in their digestive tract. Fish (n = 110) were randomly sampled from freshwater and marine habitats in Israel. Ten different fish species sampled from freshwater habitats (lake, rivers and fish ponds), and one marine species, were found to carry *V. cholerae*. The fish intestine of *Sarotherodon galilaeus* harboured ca. 5×10^3^ *V. cholerae* cfu per 1 gr intestine content—high rates compared with known *V. cholerae* cfu numbers in the bacteria's natural reservoirs. Our results, combined with evidence from the literature, suggest that fish are reservoirs of *V. cholerae*. As fish carrying the bacteria swim from one location to another (some fish species move from rivers to lakes or sea and *vice versa*), they serve as vectors on a small scale. Nevertheless, fish are consumed by waterbirds, which disseminate the bacteria on a global scale. Moreover, *V. cholerae* isolates had the ability to degrade chitin, indicating a commensal relationship between *V. cholerae* and fish. Better understanding of *V. cholerae* ecology can help reduce the times that human beings come into contact with this pathogen and thus minimize the health risk this poses.

## Introduction

Cholera, a life threatening diarrhoeal disease, still kills thousands annually and remains one of the few bacterial diseases known for its pandemicity. Of more than 200 O-antigen serogroups so far identified among *V. cholerae* isolates, only two serogroups, O1 and O139, are known to cause epidemics and pandemics [1]. Non-O1/non-O139 strains have not been found to be involved in epidemic cholera, but they are associated with non-Ol/non-O139 *V. cholerae* gastroenteritis. Although rare, non-Ol/non-O139 *V. cholerae* gastroenteritis can cause septicaemic infections and even prove lethal. The predominant symptoms associated with this illness are diarrhoea, abdominal cramps, and fever, together with vomiting and nausea and the appearance of blood and mucus in the infected individual's stools [2]. *V. cholerae* O1, O139 and non-O1/O139 comprises a single taxonomic species and their environmental habitats are likely to reveal great similarities [3].

*Vibrio cholerae*, the causative agent of cholera, is a natural inhabitant of aquatic environments, but despite intensive efforts its ecology is still poorly understood [4]. Colwell and colleagues [5]–[7] showed that *V. cholerae* proliferates while attached to or associated with eukaryotic organisms, particularly copepods (Crustacea). We found that chironomids (non-biting midges, Diptera) also serve as an intermediate host reservoir for *V. cholerae* [8]–[13]. Recently, we put forward a novel hypothesis [14] suggesting that both copepods and chironomids are dispersed by migratory birds which consume them (endozoochory) or carry them externally (epizoochory), thus distributing the bacteria among water bodies located on and between continents [14].

Here we suggest that fish also act as important reservoirs and vectors of *V. cholera*e. Indeed, cholera was associated with the consumption of salt fish, sardines, dried fish and other fish diet in different parts of the world [15]–[20]. Fish have been implicated in cholera cases in the past, but to the best of our knowledge no environmental survey of the presence of *V. cholerae* in fish has been performed so far.

In this report we show that fish of various species and habitats contain *V. cholerae* in their digestive tract. We suggest that fish serve as intermediate vectors of *V. cholerae* since they create a link in the food chain between chironomids and copepods on the one hand and waterbirds on the other [14]. As such, they are likely to pose a health risk to humans who consume them.

## Results

A total of 14 fish species were sampled from freshwater habitats of which 10 species (71%) were positive for the presence of *V. cholerae* non-O1/O139 in their intestine (Tables 1 and S1). Of the fish species that were sampled from fish ponds, 87% were positive for *V. cholerae*. *V. cholerae* was found in 60% of the fish species sampled in the Sea of Galilee and 50% of the fish species sampled in rivers. In contrast, only one of the 44 fish species (2.3%) sampled in the Mediterranean Sea was positive for *V. cholerae* (Tables 1 and S1). In most cases, the bacteria were isolated directly from the epithelial intestine without any enrichment.

**Table 1:** Fish species found positive for *V. cholerae* presence.

Habitat	Fish species (common name)	Location, Sampling date	n	Isolate code name
Fish pond	*Astatotilapia flaviijosephi* (Josephus cichlid)	Nir David, northern Israel, December 2007	1	5ASFW27
	*Ctenopharyngodon idella* (Grass carp, white-amur)	Atlit, northern Israel, November 2008	3	9AM2ME54, 9AM3BE225, 9AM4LE157
	*Cyprinus carpio* (Common carp)	Atlit, northern Israel, November 2008	1	9CALE136
	*Mugil cephalus* (Flathead grey mullet)	Nahalal, North Israel, October 2008	2	7AN1CW74, 7AN3P49
	*Sarotherodon galilaeus* (Galilee st. Peter's fish)	Kfar Rupin, eastern Israel, November 2008	8	10AN1C27, 10AN2E22, 14AN1C82, 14AN2C65, 14AN3C101, 14AN4C60, 14AN5P40, 14AN6C36
	*Tilapia* sp.	Nir David, northern Israel, November 2007	2	4AN3W61, 5AN2W73
		Nahalal, North Israel, October, 2008	2	7AN1CW74, 7AN3P49
	*Tilapia zillii* (Common St. Peter's fish)	Fish pond, northern Israel, November 2007	2	1AN3W52, 1AN4P53
The Sea of Galilee	*Barbus canis* (Large scale barbel)	November 2008	1	10BGK3E44
	*Barbus longiceps* (Longhead barbel)	November 2008	1	10BAR1P91
	*Mugil cephalus* (Flathead grey mullet)	December 2008	2	14BR4C1VC, 14BR5C88VC
River	*Oreochromis aureus* (Jordan St. Peter's fish)	Asi stream, Nir David, northern Israel, February 2009	1	16g1P73
Mediterranean Sea	*Myripristis murdjan* (Blotcheye soldierfish)	Akko, December 2008	1	12e1E8

None of *V. cholerae* strains isolated from the fish and examined in this study contained the genes for *ctxA*, *zot*, *tcpA*, *tcpI* or for non-O1 heat-stable enterotoxin (*stn/sto*). All isolates possessed *toxR* and the structural gene *hapA*, which is responsible for soluble haemagglutinin/protease (HA/P) production (Table 2). The *hlyA*, *hapA*, *ompU*, *vcsC2*, *vcsN2*, *vspD* and *vcsV2* genes were found in 84%, 84%, 60%, 48%, 34%, 50% and 52% of the strains, respectively (Table 2). The genotypes were found in various combinations (Table 2). Thirty-two percent of the strains were positive for all four loci of the type III secretion system (TTSS) cluster (*vcsC2*, *vcsN2*, *vspD* and *vcsV2* genes). Ninety-four percent of the strains that were positive for the TTSS genes were also positive for *hlyA* gene encoding the haemolysin toxin (Table 2). No correlation was found between the fish species and the genotypes of the isolated *V. cholerae* strains. Strains that were positive for both TTSS and *hlyA* genes were isolated from various fish species that were sampled from all types of habitats; *Cyprinus carpio* and *Sarotherodon galilaeus* (fish pond); *Mugil cephalus* (Sea of Galilee); *Oreochromis aureus* and *Astatotilapia flaviijosephi* (river); *Myriprsitis murdjan* (Mediterranean Sea).

**Table 2:** Genotypic traits of *V. cholerae* strains examined in this study (n = 50).

No. of strains	Presence or absence of potential virulent genes^a^			
	TTSS^b^	*ompU*	*hlyA*	*hapA*
7	+	+	+	+
8	+	−	+	+
1	+	−	−	−
7	±	+	+	+
2	±	−	+	+
1	±	+	−	+
1	±	−	−	−
12	−	+	+	+
2	−	−	+	+
2	−	+	+	−
2	−	−	+	−
2	−	−	−	+
1	−	+	−	+
2	−	−	−	−

a Presence (+) or absence (−) of potential virulence genes is shown.

b PCR-based detection of the TTSS cluster by the presence of *vcsC2*, *vcsN2*, *vspD*, and *vcsV2*. Symbols are (+) for presence, (−) for absence, (±) for presence of some but not all of the genes tested.

All the examined strains were *ctxA*, *zot*, *tcpA*, *tcpI* and *stn/sto* negative and *toxR* positive.

*V. cholerae* numbers in fish intestine content were estimated in two fish species: *Sarotherodon galilaeus* (Galilee St. Peter's fish) sampled from a fish pond and *Mugil cephalus* (Flathead grey mullet) sampled from the Sea of Galilee (Table 3). *V. cholerae* cfu (colony forming units) in the fish intestine of *Sarotherodon galilaeus* was found to be ca. 5×10^3^ per one gram intestine content. This was 35 times more than *V. cholerae* content in *Mugil cephalus* (Table 3). *V. cholerae* isolates from all the fish species showed the ability of chitin degradation.

**Table 3:** Estimation of *V. cholerae* cfu in fish intestine content.

Fish species	Sampling location	n	*V. cholerae* cfu/g intestine content±SE
*Sarotherodon galilaeus* (Galilee St. Peter's fish)	Fish pond	6	4.8×10^3^±7.1×10^2^
*Mugil cephalus* (Flathead grey mullet)	Sea of Galilee	2	1.4×10^2^±5.0×10^1^

Sampling date, December 2008.

## Discussion

Copepods and chironomids, both natural reservoirs of *V. cholerae*, are abundant in fresh and marine water ecosystems and are consumed by fish. Here we showed that *V. cholerae* inhabits the intestines of various fish species sampled from freshwater habitats and one species sampled from a marine habitat. In most cases, the bacteria were isolated without the use of any enrichment methods, demonstrating that *V. cholerae* is abundant in some fish species. Thus, we demonstrated that fish serves as intermediate reservoirs of *V. cholerae* in various aquatic ecosystems. We suggest that the bacteria are introduced into the fish intestine via their invertebrate prey (Table S2).

The fish intestine of *Sarotherodon galilaeus* harboured ca. 5×10^3^ *V. cholerae* cfu per 1 gr intestine content. This is one magnitude higher than what was found in chironomid egg masses [12]. As most (99%) of the *V. cholerae* inhabiting the egg mass were found in the viable but non-culturable (VBNC) state [12], this may well be the case here. However, the sampling methods employed in this study may not distinguish actual colonization from surface contamination, so the numbers may not reflect the actual colonized bacteria.

We found evidence from the literature correlating the source of cholera disease with fish but no study actually surveyed the presence of the bacteria in the fish. It was postulated that cholera endemicity in India was due to hilsa fish [15]. Cholera has been associated with consumption of raw fish in several countries in different continents [16]–[20]. In a case of cholera, caused by *V. cholerae* O1, reported in Berlin, the patient had most likely been infected while handling and preparing fish imported from Nigeria [21]. A tropical fish tank was associated with *V. cholerae* that caused a wound [22]. Other reports that connect *V. cholerae* to fish include the isolation of *V. cholerae* from ayu fish in Japan [23], from fish tank water in Hong Kong [24], and from aquarium water from fish imported from Thailand and Sri Lanka to Czechoslovakia [25].

Although there is one report pointing to *V. cholerae* as a possible cause of death in ayu fish in Japan [23], fish actually may benefit from *V. cholerae* that inhabit their intestine. Strains of *V. cholerae* secrete extracellular enzymes such as proteases [9] and chitinases [26]. These enzymes may facilitate the use of macromolecules that the fish digest. Chitin is the most abundant polysaccharide in nature after cellulose and it is a source of both carbon and nitrogen. This insoluble material is recycled mainly by chitinolytic bacteria, including members of the family *Vibrionaceae* [26], [27]. Chitin is the main component of the exoskeletons of crustaceans (copepods) and insects (chironomids). Thus, *V. cholerae* in fish intestine may help fish to digest their prey. Indeed, *V. cholerae* isolates from all fish species in the current study showed the ability to degrade chitin, indicating a commensal relationship between *V. cholerae* and fish.

Our results, combined with evidence from the literature, suggest that fish are possible reservoirs of *V. cholerae*. As fish carrying the bacteria swim from one location to another (some fish species move from rivers to lakes or sea and *vice versa*), they serve as vectors on a small scale. Nevertheless, fish are consumed by waterbirds, which disseminate the bacteria on a global scale [14].

As far as we know, ours is the first survey of fish serving as reservoir of *V. cholerae*. So far, we have succeeded in isolating from fish only the non-O1/O139 serogroups (the last cholera outbreak in Israel, only two cases, was reported in 1989). Nevertheless, non-O1/O139 serogroups are likely to share the same environmental habitats as O1/O139 serogroups [3].

*V. cholerae* non-O1/O139 serogroups have been reported to cause human disease such as sporadic outbreaks of watery diarrhoea and inflammatory enterocolitis [28]–[31]. Evidence suggests that virulence factors other than cholera toxin might be crucial in the pathogenesis of *V. cholerae* non-O1/O139 induced diarrhoea. Type III secretion system (TTSS), which has been associated with pathogenic mechanisms in a wide variety of bacteria, is now recognized as potential virulence factors of non-O1, non-O139 strains as well as *V. parahaemolyticus* [31]–[33]. Thirty-two percent of the strains in our study were found to contain the TTSS gene cluster, indicating the virulence potential of the fish isolates (Table 2). Debellis et al. [34] demonstrated that haemolysin, a virulence factor present in non-O1/O139 strains of *V. cholerae*, forms anion channels on the apical membrane of enterocytes, thus promoting chloride secretion from intact human intestinal mucosa. Indeed, strains of non-O1/O139 *V. cholerae* isolates from hospitalized diarrhoeal patients in Kolkata, India, were positive only for the *hlyA* gene, and negative for all other known pathogenic genes of *V. cholerae* [31]. In the current study 84% of the *V. cholera*e isolates from fish were found positive to *hlyA* gene encoding the haemolysin toxin (Table 2).

The source of ca. 70% of the fish consumed by humans as food items are grown by fish farming. Fish yield in intensive fish ponds may reach ca. 5 tons of fish per ha. In the current study seven out of eight fish species sampled from fish ponds proved positive for *V. cholerae*. As fish are a staple food in the global human diet, this creates the vital need to assess the health risk posed by their potential infection with *V. cholerae* from O1/O139 or non-O1/O139 serogroups. More research is needed to investigate the possibility that unsolved diarrhoea cases of patients who reported eating or handling fish were caused by non-O1/O139 serogroups of *V. cholerae*. Moreover, a survey, to isolate pathogenic O1 and O139 serogroups strains from fish should be performed in an endemic area of the disease.

*V. cholerae* cannot be eradicated: it is a part of the normal flora of the surface water of our planet. Thus, only a fuller understanding of its ecology can help reduce the times that human beings come into contact with this pathogen, thus minimizing the health risk this poses.

## Methods

### Ethics Statement

All the fish in the current study were obtained in different locations from fishermen selling fresh fish for consumption.

### Sampling Sites

Fish were randomly sampled from several freshwater habitats in Israel: lake (the Sea of Galilee), rivers (Asi and Nir David, northern Israel), fish ponds (Nir David, Nahalal, Atlit, northern Israel; Kfar Rupin, eastern Israel) as well as from the Mediterranean Sea (Tables 1 & S1).

### Sampled Fish

The species of fish that were sampled are listed in Table 1 and in Table S1. The collected fish were brought into the lab and samples were taken from their intestines. The samples were treated for isolation and identification of *V. cholerae* as specified below.

### Isolation and Identification of *V. cholerae*

Middle or lower intestine contents were either inoculated into alkaline peptone water (APW) containing peptone (1%, wt/vol) and NaCl (1%, wt/vol) or directly streaked on TCBS (Thiosulfate Citrate Bile Sucrose agar, Difco) without enrichment. In the case of enrichment, the tubes were incubated at 37°C without shaking for 6–18 h, and then streaked on TCBS and incubated overnight at 37°C. In case of direct intestine examination, the intestine epithelia were scratched with a bacteriological sterile needle and then immediately streaked on TCBS agar. Yellow colonies from TCBS medium that were suspected as *V. cholerae* were subcultured onto LB agar, and then tested for oxidase (1% tetramethyl-p-phenylenediamine; Sigma) and subjected to the string test (0.5% sodium deoxycholate; Sigma). The identity of the isolates with positive results in the above tests was further verified by multiplex PCR assay in accordance with Nandi et al. [35]. This multiplex PCR identifies the presence of *ompW*, a gene of an outer membrane protein, specific to *V. cholerae*, and *ctxA*, the gene of cholera toxin. All isolates identified as *V. cholerae* were further examined to determine whether they were members of the O1 and O139 serogroups by slide agglutination with use of two specific antisera; (1) a poly antiserum specific for O1 surface antigen Inaba or Ogawa (Difco), and (2) an antiserum specific for O139 surface antigen (Ministry of Health, Israel). The presence of toxin genes (in addition to *ctxA*) *zot*, *stn*/*sto*, *hlyA*, *hapA tcpA* and *ompU* was determined in all *V. cholerae* strains. In addition, the strains were examined for the presence of regulatory genes for TCP expression (*tcpI*) and the central regulatory protein (*toxR*). The primers used in this study and the PCR procedures are described in Halpern et al. [11]. The *vcsC2*, *vcsN2*, *vspD* and *vcsV2* genes were used as target loci for PCR-based amplification for the detection of the TTSS cluster in the different strains. The primers and the PCR procedures for the detection of the TTSS cluster are described in Chatterjee et al. [31] and Dziejman et al. [32]. The strains were maintained in LB with 30% glycerol (−80°C).

### Enumeration of *V. cholerae*

The number of *V. cholerae* per one gram of fish intestine content was estimated in two fish species: *Sarotherodon galilaeus* (Galilee St. Peter's fish) and *Mugil cephalus* (Flathead grey mullet). Samples were weighed and then added to 1 ml sterile saline. These were vortexed and diluted, and 0.1 ml from each dilution was spread onto TCBS agar and incubated overnight at 37°C. Yellow colonies from TCBS medium suspected as *V. cholerae* were counted and subcultured onto LB agar. The colonies were then identified as described above.

### Chitin Degradation Assay

Colloidal chitin was prepared from practical grade chitin (Sigma) from crab shells as described by Hsu and Lockwood [36] with slight modifications. Forty grams of practical grade chitin (Sigma) from crab shells were dissolved in 400 ml of concentrated HCl. The chitin was precipitated as a colloidal suspension by adding it slowly to 2 litres of water at 5–10°C. The suspension was collected by filtration with suction on Whatman #1 paper. The precipitated chitin was dialysed against tap water for 12 h, and the pH was adjusted to 7.0 with KOH. The chitin agar was prepared using a sufficient volume of colloidal chitin suspension to give 4 g of chitin. These were mixed with the following mineral salts: (NH_4_)_2_SO_4_ (2.0 g), Na_2_HPO_4_ (1.1 g), KH_2_PO_4_ (0.7 g), MgSO_4_*7H_2_O (0.2 g), FeSO_4_ (1.0 mg), MnSO_4_ (1.0 mg) and 20 g of agar. Distilled water was added to final volume of 1.0 L. The medium was mixed thoroughly and autoclaved. *V. cholerae* strains that were isolated from fish were cultured on this medium and incubated at 37°C. A clear zone around the bacterial colonies showed the presence of chitinase activity.

## Supporting Information

Table S1 Fish species found negative for *V. cholerae* presence.
(0.09 MB DOC)

Table S2 Food habits of fish species found positive for *V. cholerae* presence.
(0.04 MB DOC)
