# Supplementary material for: Fish as Reservoirs and Vectors of Vibrio cholerae
Source: PLoS One. 2010 Jan 6;5(1):e8607. doi: 10.1371/journal.pone.0008607 (PMC2797615; doi:10.1371/journal.pone.0008607)
Supplement: Table S1 — Fish species found negative for V. cholerae presence. (0.09 MB DOC) [file pone.0008607.s001.doc]

**Supplementary Data**

**Table S1**. Fish species found negative for *V. cholerae* presence.

| **Habitat** | **Fish species (common name)** | **Sampling date** | **n** |
| --- | --- | --- | --- |
| Mediterranean Sea, Ako | *Alepes djedaba* (Shrimp's scad) | November, 2007 | 1 |
|  | *Argyrosomus regius* (Meagre) | January, 2009 | 1 |
|  | *Balistes carolinensis* (Gray triggerfish) | November, 2007 | 1 |
|  | *Caranx crysos* (Blue runner) | February, 2009 | 2 |
|  | *Citharus linguatula* (Spotted flounder) | November, 2007 | 1 |
|  | *Dentex dentex* (Common dentex) | November, 2007 | 1 |
|  | *Dicentrarchus labrax* (European seabass) | February, 2009 | 2 |
|  | *Dicentrarchus punctatus* (Spotted seabass) | March, 2009 | 1 |
|  | *Diplodus capensis* (Cape white seabream) | January, 2009 | 1 |
|  | *Diplodus cervinus* (Zebra sea bream) | November, 2007 | 1 |
|  | *Diplodus puntazzo* (Sharpsnout sea bream) | November, 2007 | 1 |
|  | *Diplodus sargus* (White sea bream) | March, 2009 | 5 |
|  | *Euthynnus alletteratus* (Little tunny) | November, 2007 | 1 |
|  | *Hemiramphus far* (Halfbeak) | November, 2007 | 2 |
|  | *Lithognathus mormyrus* (Striped sea bream) | November, 2007 | 1 |
|  |  | March, 2009 | 3 |
|  | *Liza aurata* (Golden grey mullet) | March, 2009 | 3 |
|  | *Liza ramada* (Thinlip grey mullet) | February, 2009 | 2 |
|  | *Mullus barbatus* (Red mullet) | November, 2007 | 1 |
|  | *Mullus surmuletus* (Striped red mullet) | December, 2008 | 2 |
|  | *Oblada melanura* (Saddled bream) | March, 2009 | 2 |
|  | *Pagellus acarne* (Axillary sea bream) | December, 2008 | 1 |
|  | *Pagellus erythrinus* (Common pandora) | November, 2007 | 2 |
|  |  | March, 2009 | 2 |
|  | *Pagrus coeruleostictus* (Blue-spotted sea bream) | November, 2007 | 1 |
|  |  | February, 2009 | 2 |
|  | *Pomadasys incisus* (Bastard grunt) | November, 2007 | 1 |
|  | *Sardinella aurita* (Round sardinella) | November, 2007 | 1 |
|  | *Sarpa salpa* (Salema) | March, 2009 | 1 |
|  | *Saurida undosquamis* (Brushtooth lizardfish) | November, 2007 | 2 |
|  |  | December, 2008 | 1 |
|  | *Scomberomorus commerson* (Narrow-barred Spanish mackerel) | November, 2007 | 1 |
|  | *Siganus rivulatus* (Marbled spinefoot, Rabbitfish) | November, 2007 | 2 |
|  |  | December, 2008 | 1 |
|  | *Sillago sihama* (Silver sillago, Whiting) | November, 2007 | 1 |
|  | *Sparus aurata* (Gilt-head sea bream) | March, 2009 | 2 |
|  | *Sphyraena sphyraena* (Barracuda) | November, 2007 | 1 |
|  |  | December, 2008 | 1 |
|  | *Trachinotus ovatus* (Derbio, Pompano) | November, 2007 | 1 |
|  | *Trachurus mediterraneus* (Mediterranean horse-mackerel) | November, 2007 | 1 |
| Mediterranean Sea, Haifa | *Boops boops* (Bogue) | December, 2008 | 1 |
|  | *Hemiramphus far* (Halfbeak) | April, 2009 | 4 |
|  | *Lagocephalus spadiceus* (Puffer, Blaasop) | December, 2008 | 1 |
|  | *Lagocephalus suezensis* (Lagocephalus suezensis) | December, 2008 | 1 |
|  | *Lepidotrigla* sp. | December, 2008 | 1 |
|  | *Nemipterus randalli* (Randall's threadfin bream) | December, 2008 | 1 |
|  | *Pagellus acarne* (Axillary sea bream) | December, 2008 | 1 |
|  | *Pagellus erythrinus* (Common pandora) | December, 2008 | 1 |
|  | *Plotosus lineatus* (Striped eel catfish) | December, 2008 | 1 |
|  | *Saurida undosquamis* (Brushtooth lizardfish) | December, 2008 | 1 |
| Fish pond, north Israel | *Clarias lazera* (North African catfish) | November, 2007 | 1 |
| Sea of Galilee | *Capoeta damascina* (Damascus barbel) | January, 2008 | 2 |
|  | *Liza ramada* (Thinlip mullet) | January, 2008 | 2 |
| Stream, north Israel | *Poecilia latipinna* (Sailfin Molly) | February, 2009 | 5 |
