# Supplementary material for: Fish as Reservoirs and Vectors of Vibrio cholerae
Source: PLoS One. 2010 Jan 6;5(1):e8607. doi: 10.1371/journal.pone.0008607 (PMC2797615; doi:10.1371/journal.pone.0008607)
Supplement: Table S2 — Food habits of fish species found positive for V. cholerae presence. (0.04 MB DOC) [file pone.0008607.s002.doc]

**Supplementary Data**

**Table S2.** Food habits of fish species found positive for *V. cholerae* presence.

| **Nutrition** | **Fish species** |
| --- | --- |
| Females feed mainly on chironomid larvae, oligochaetes and amphipods while the diet of larger males consists mainly of gastropods. | *Astatotilapia flaviijosephi* |
| Feeds on invertebrates and small fish. | *Barbus canis* |
| In the Sea of Galilee the main food items are chironomid larvae and oligochaetes, while gastropods form only a minor part of the diet. | *Barbus longiceps* |
| Feeds on higher aquatic plants and submerged grasses; takes also detritus, insects and other invertebrates. | *Ctenopharyngodon idella* |
| Omnivorous, feeding mainly on aquatic insects, crustaceans, annelids, mollusks, weed and tree seeds, wild rice, aquatic plants and algae; mainly by grubbing in sediments. | *Cyprinus carpio* |
| Feeds on zooplankton as larvae, detritus, micro-algae and benthic organisms as juvenile and adult fish. | *Mugil cephalus* |
| Feeds mainly on plankton such as crab larvae. | *Myripritis murdjan* |
| Feeds on phytoplankton and small quantities of zooplankton. Young fish have a more varied diet, which includes large quantities of copepods and cladocerans. | *Oreochromis aureus* |
| Feeds on algae and fine organic debris. | *Sarotherodon galilaeus* |
| Herbivorous, feeds on water plants and epiphyton, and some invertebrates. | *Tilapia zillii* |
